# Dendritic cell–related gene signature in pancreatic cancer stratifies patient subtypes and implicates a KCTD14–TNF signaling axis

**DOI:** 10.3389/fimmu.2025.1665906

**Published:** 2025-09-25

**Authors:** Yuxin Liang, Yuheng Gu, Zilong Zhang, Deyuan Zhong, Hongtao Yan, Yuhao Su, Yahui Chen, Fei Wang, Zhengwei Leng, Xiaolun Huang

**Affiliations:** ^1^ Liver Transplantation Center and HBP Surgery, Sichuan Clinical Research Center for Cancer, Sichuan Cancer Hospital & Institute, Sichuan Cancer Center, School of Medicine, University of Electronic Science and Technology of China, Chengdu, China; ^2^ Clinical Medical College, Southwest Medical University, Luzhou, China; ^3^ Hepatic Surgery Center, Clinical Medical Research Center of Hepatic Surgery at Hubei Province, Hubei Key Laboratory of Hepato-Pancreatic-Biliary Diseases, Tongji Hospital, Tongji Medical College, Huazhong University of Science and Technology, Wuhan, Hubei, China; ^4^ Center for Natural Products Research, Chengdu Institute of Biology, Chinese Academy of Sciences, Chengdu, China

**Keywords:** pancreatic ductal adenocarcinoma, dendritic cells, Kctd14, prognosis, signature, TNF signaling

## Abstract

**Background:**

Pancreatic cancer (PC) is characterized by a profoundly immunosuppressive tumor microenvironment and poor prognosis. Dendritic cells (DCs) are pivotal for antigen presentation and T-cell activation, yet their prognostic and mechanistic roles in PC remain incompletely defined.

**Methods:**

This study performed weighted gene co-expression network analysis (WGCNA) on transcriptomic data from The Cancer Genome Atlas (TCGA) and two Gene Expression Omnibus cohorts (GSE62165, GSE85916) to identify DC–related gene modules. Consensus clustering based on these modules stratified patients into two immune phenotypes. A four-gene DC–related risk score (DCRS) was constructed using LASSO-Cox regression and validated in independent cohorts. Single-cell RNA sequencing data from 25 PC samples (GSE242230) were analyzed through cell clustering analysis, cell-cell communication analysis, and pathway-specific analysis. Functional assays following *KCTD14* knockdown in CAPAN-1 and PANC-1 cell lines assessed its impact on proliferation, migration, invasion, and TNF signaling.

**Results:**

WGCNA identified 130 overlapping DC–related genes enriched in immune pathways. Two DC–related patient clusters exhibited distinct overall survival (OS) (P < 0.05). The DCRS robustly stratified patients into high- and low-risk groups in both TCGA training and validation sets. DCRS demonstrated good predictive potential for OS and there is a significant difference in OS between the two groups of patients (*P* < 0.05). Single-cell analysis revealed *KCTD14* enrichment in malignant epithelial cells and predicted its interaction with DCs via the TNF-TNFRSF1A axis. *In vitro*, *KCTD14* knockdown significantly reduced PC cell proliferation, colony formation, migration, and invasion, and downregulated TNF-α and TNFRSF1A expression (P < 0.01).

**Conclusion:**

We identified a novel DC–related gene signature that stratifies PC patients by prognosis and highlights KCTD14 as a novel immunomodulatory oncogene acting through the TNF-TNFR1 axis. Our findings provide a foundation for integrating DCRS into clinical risk assessment and for pursuing KCTD14/TNFR1-targeted therapies to overcome DC-mediated immune suppression in pancreatic cancer.

## 1 Introduction

Pancreatic cancer (PC) remains among the most aggressive malignancies, with a 5-year survival rate of less than 10% and limited therapeutic options (1–3). Despite advances in surgical techniques and systemic therapies, most patients are diagnosed at an advanced stage when curative treatment is no longer feasible (4). Postoperative recurrence, drug resistance, and persistent low response to treatment are still problems in the treatment of PC (5). Therefore, the development of reliable indicators for early detection and risk stratification represents an urgent unmet clinical need. To date, numerous studies have sought biomarkers for PC, ranging from subcellular organelle dysfunction, tumor-immune interactions and epigenetic alterations (6–8). Nevertheless, clinically validated early diagnostic markers, therapeutic targets, and risk assessment algorithms for PC remain lacking.

The tumor immune microenvironment of PC is characterized by chronic inflammation coupled with profound immunosuppression, which together impede the infiltration and function of antitumor effector cells (9, 10). In particular, dendritic cells (DCs), the professional antigen-presenting cells that bridge innate and adaptive immunity, are notably scarce or functionally impaired in PC (11, 12). DCs normally capture tumor antigens and migrate to lymphoid tissues to prime T and B lymphocytes, driving antitumor immune responses (13). Insufficient DCs numbers or maturation results in poor activation of cancer-specific T cells. Indeed, studies in PC models have shown that tumor-derived TNF-α signaling through TNF receptor 1 (TNFR1) causes apoptosis of DCs, thereby depleting intratumoral DCs and weakening immunity (9, 14). These findings highlight the pivotal role of DCs and the TNF-TNFR1 axis in shaping the anti-tumor immune landscape of PC.

In the present study, we aimed to develop a DC–related gene signature for PC prognosis and to elucidate the molecular crosstalk between tumor cells and DCs. Leveraging bulk transcriptomic datasets from multiple pancreatic ductal adenocarcinoma (PDAC) cohorts, we stratified patients into two immune phenotypes and constructed a DC-related gene signature. We then interrogated single-cell RNA-sequencing data to pinpoint key gene of DC-tumor interactions. Finally, we performed *in vitro* assays to assess its effect on tumor cell proliferation, migration, invasion, and downstream signaling pathways. Our integrative approach seeks to uncover novel biomarkers for early diagnosis and risk assessment and to identify potential targets for immunomodulatory therapy in PC.

## 2 Materials and methods

### 2.1 Data acquisition

Transcriptome profiles and corresponding clinicopathological information for PDAC were collected from The Cancer Genome Atlas (TCGA)-pancreatic adenocarcinoma (PAAD) and two independent Gene Expression Omnibus (GEO) cohorts (GSE62165 and GSE85916). To minimize inter-dataset variability, batch correction was implemented using the “ComBat” function within the *sva* R package (15, 16).

### 2.2 DC-related gene and consensus clustering analysis

Immune cell infiltration in TCGA-PAAD, GSE62165, and GSE85916 samples was quantified using the TIME method. Weighted gene co-expression network analysis (WGCNA) was then employed to construct gene modules correlated with DC infiltration across all cohorts. Intersection of module genes via Venn diagrams yielded a PDAC-specific DC–related gene set. Based on these genes, unsupervised consensus clustering of TCGA-PAAD samples based on this gene set was performed with the *ConsensusClusterPlus* package. Differentially expressed genes (DEGs) between clusters were determined by the *limma* package, with significance thresholds of adjusted *P*< 0.05.

### 2.3 Functional enrichment analysis

To characterize the biological significance of candidate genes, Gene Ontology (GO) and Kyoto Encyclopedia of Genes and Genomes (KEGG) pathway enrichment analyses were conducted. In addition, Gene Set Enrichment Analysis (GSEA) was performed to evaluate pathway–level enrichment (17). All analyses were performed using the *clusterProfiler* package.

### 2.4 Identification of the DC-related risk score for HCC

Patients from the TCGA-PAAD dataset were first stratified into two subtypes by consensus clustering. Candidate genes were obtained from the intersection of DEGs between subtypes and those between tumor and adjacent non-tumor samples. The TCGA-PAAD cohort was randomly divided into training and validation sets using the *caret* package. In the training cohort, prognostic genes were screened by univariate Cox regression and subsequently refined with least absolute shrinkage and selection operator (LASSO) regression using the *glmnet* package (18). Risk scores were generated as a weighted sum of gene expression values, with Cox coefficients as weights. Patients were dichotomized into high- and low-risk groups according to the median risk score, and the model’s predictive performance was further confirmed in the TCGA validation set.

### 2.5 Single-cell RNA sequencing analysis

Single-cell RNA sequencing data from 25 PDAC patients were acquired from the GEO database (GSE242230) for comprehensive analysis. The gene expression matrix was processed using the *Seurat* package, with stringent normalization and filtering criteria applied: a minimum of 200 genes per cell, no more than 20% mitochondrial genes, and at least 5% ribosomal genes (19). Subsequently, nonlinear dimensionality reduction, cell clustering, and visualization were performed to delineate the expression profiles of target genes across various cell types.

### 2.6 Cell clustering analysis, cell-cell communication analysis, and Pathway-specific analysis

Cell clustering analysis was conducted by t-distributed stochastic neighbor embedding (t-SNE) dimensionality reduction with a resolution parameter of 0.5. Clusters were visualized using the *Seurat* and *ggplot2* packages (20), with custom color palettes applied for enhanced visualization. Cell type annotation was conducted through manual curation based on established marker genes and cluster characteristics. Gene expression visualization was performed using feature plots and dot plots to examine the expression patterns of DC markers including CLEC9A, XCR1, BATF3, CADM1, CD1C, FCER1A, CLEC10A, LILRA4, CLEC4C, and IRF7 across different clusters. Density plots for genes of interest (*CD81*, *KCTD14*, *GBP1*, and *MYEOV*) were generated using the *Nebulosa* package to visualize spatial expression patterns. For cell-cell communication analysis, epithelial cells were stratified based on *KCTD14* expression status into KCTD14-positive and KCTD14-negative subgroups. Cell-cell communication networks were analyzed using the *CellChat* package with the human CellChatDB database. A subset of 3,000 randomly selected cells was used for computational efficiency. We computed interaction probabilities (cell threshold = 0; pathway threshold = 1), visualizing networks via circular plots. Pathway-specific analyses (TNF, TGFβ, SPP1, SEMA4, and MIF) included heatmaps, ligand–receptor contributions, and top interacting pairs. Interactions with *P* < 0.01 were deemed significant. All visualizations and statistical analyses were performed using R software with appropriate packages including *Seurat*, *ggplot2*, *Nebulosa*, *CellChat*, and *ktplots*.

### 2.7 Cell culture and cell transfection

Human pancreatic ductal epithelial cells (H6C7) and pancreatic cancer cell lines (CAPAN-1, PANC-1) were obtained from the Cell Bank of the Chinese Academy of Sciences (Shanghai, China). Cells were cultured in DMEM supplemented with 10% fetal bovine serum (FBS) and 1% penicillin-streptomycin at 37°C with 5% CO_2_ (21). For *KCTD14* knockdown, cells at 60–70% confluence were transfected with small interfering RNAs (siRNAs) targeting KCTD14 (si−KCTD14−1, si−KCTD14−2, si−KCTD14−3) or non−targeting control siRNA using Lipofectamine 3000 (Invitrogen). After 48 hours, cells were harvested for downstream assays.

### 2.8 Quantitative real−time polymerase chain reaction

Total RNA was extracted with TRIzol reagent (Invitrogen) and reverse−transcribed using the PrimeScript RT kit (Takara). qRT−PCR was conducted with SYBR qPCR Master Mix (Vazyme, China). Primer sequences were as follows:

KCTD14 (Forward): 5′−AGCAAACATGAACAGTAGGTTA−3′,

KCTD14 (reverse): 5′−GGAATGAAGGTAAGCAAAC−3′. Relative gene expression was calculated by the 2^(^−^ΔΔCt) method with β-actin as internal reference.

### 2.9 Western blot

Cells were lysed in RIPA buffer supplemented with protease and phosphatase inhibitors (Roche). Protein concentrations were quantified by BCA assay (Pierce). Equal amounts of protein were separated on 10% SDS–PAGE gels and transferred onto PVDF membranes (Millipore). Membranes were blocked in 5% non−fat milk and incubated overnight at 4°C with primary antibodies against KCTD14, TNF−α, TNFRSF1A, TNFRSF1B, and β-actin. After washing, membranes were incubated using HRP−conjugated secondary antibodies and bands were visualized by ECL substrate (Thermo) and quantified by ImageJ (22).

### 2.10 CCK−8, colony formation, migration and invasion assays

Cell proliferation was assessed with the CCK-8 assay by seeding 3 × 10³ transfected cells per well in 96-well plates, with viability measured at 24, 48, 72, and 96 hours. Colony formation assays were performed by seeding 500 cells per well in six-well plates and allowing growth for 10–14 days before fixation and crystal violet staining. Colonies containing ≥50 cells were counted. Migration and invasion were examined using 8-μm pore Transwell chambers, with invasion assays performed in Matrigel-coated inserts. After incubation (24 h for migration, 48 h for invasion), cells on the lower membrane were fixed, stained, and quantified in five random fields (23, 24).

### 2.11 Statistical analysis

All statistical analyses were performed with R software version 4.0.1. Data were compared using Student’s *t*-test or Wilcoxon rank-sum test, as appropriate. Survival analyses were conducted with the Cox proportional hazards model and Kaplan–Meier method, with significance assessed by the log-rank test. Predictive accuracy was evaluated using time-dependent receiver operating characteristic (ROC) curves. For *in vitro* assays, all experiments were performed in three independent biological replicates, with each assay including three technical replicates unless otherwise specified. *P* value < 0.05 was considered statistically significant.

## 3 Results

### 3.1 Identification of the DC–related gene set in PC

**Figure 1:**
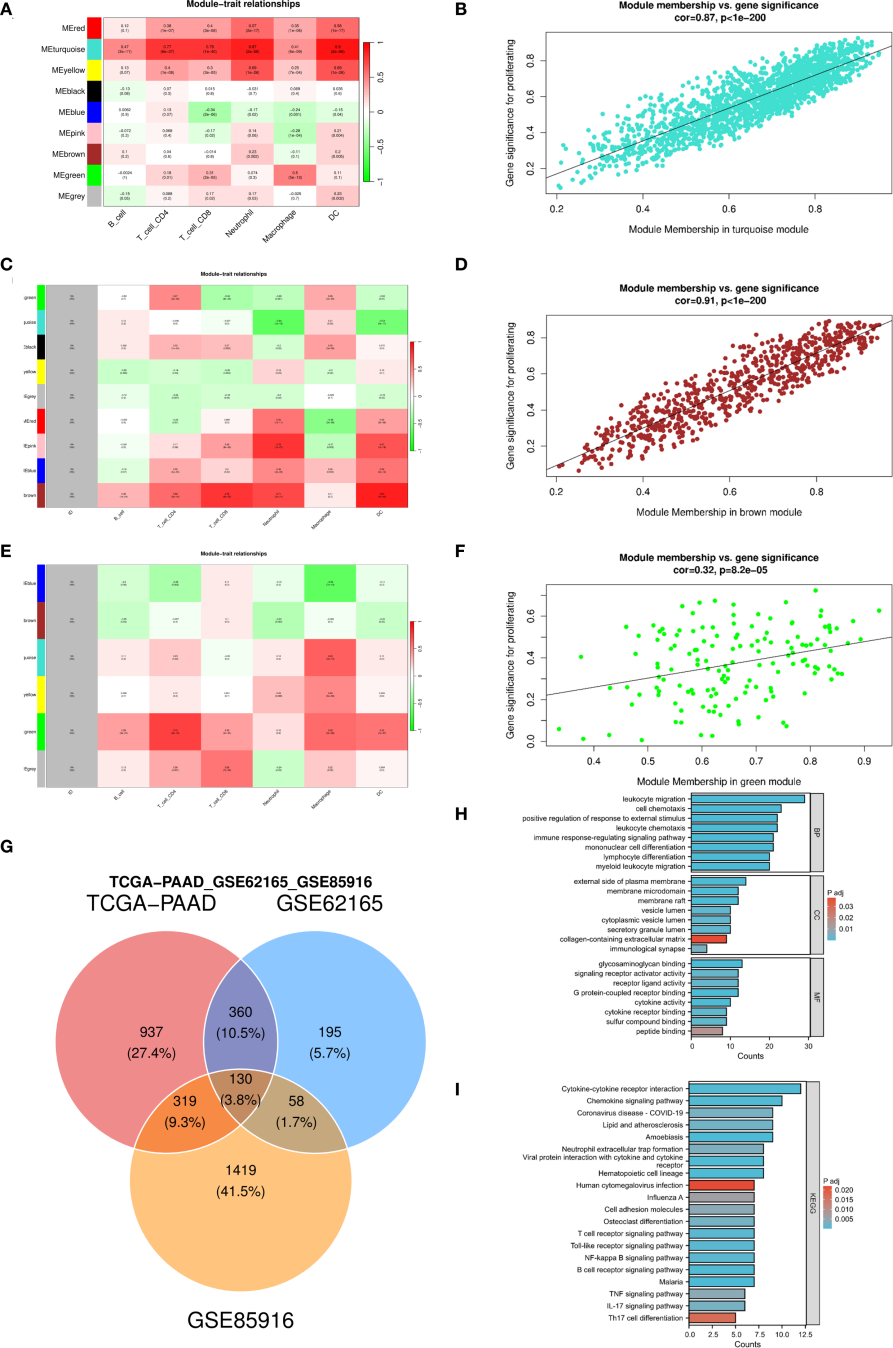
Identify DC-related gene modules and their functional enrichment across three pancreatic cancer cohorts. **(A–F)** Weighted gene co-expression network analysis identifies gene modules associated with immune cell infiltration and gene modules most strongly correlated with DCs in the TCGA-PAAD **(A, B)**, GSE62165 **(C, D)**, and GSE85916 **(E, F)** datasets separately; **(G)** Venn diagram showing the intersection of genes from the three dataset-specific modules to define a core set of DC-related genes; **(H, I)** GO enrichment analysis and KEGG pathway analysis of DC-related genes. DC, dendritic cell; TCGA, The Cancer Genome Atlas; PAAD, pancreatic adenocarcinoma; GO, Gene Ontology; KEGG, Kyoto Encyclopedia of Genes and Genomes.

WGCNA was applied to three independent PDAC cohorts to identify modules most closely associated with DC infiltration. In the TCGA cohort, the turquoise module (1746 genes) showed the strongest association with DC infiltration (correlation coefficient = 0.87, *P* < 1e-200) (**Figures 1A, B**). Similarly, in GSE62165, the brown module (743 genes) correlated most highly with DC infiltration (correlation coefficient = 0.91, *P* < 1e-200) (**Figures 1C, D**). In GSE85916, the green module (1926 genes) showed a moderate but significant association with DC infiltration (correlation coefficient = 0.32, *P* = 8.2e-5) (**Figures 1E, F**). Taking the intersection across these three modules, we identified a set of 130 DC–related genes (**Figure 1G**). GO enrichment analysis of these 130 genes revealed significant enrichment in pathways such as leukocyte chemotaxis, immune response**–**regulating signaling pathway, and lymphocyte differentiation (**Figure 1H**). KEGG enrichment analysis further revealed enrichment in pathways such as cytokine-cytokine receptor interaction, chemokine signaling pathway, T cell receptor signaling pathway, and TNF signaling pathway (**Figure 1I**). Protein–protein interaction (PPI) network construction identified *ITGAL*, *GAS7*, and *STAB1* as hub nodes with high connectivity (**Supplementary Figure S1**), suggesting their central roles in DC recruitment and activation.

### 3.2 Identification of DC–related molecular clusters

**Figure 2:**
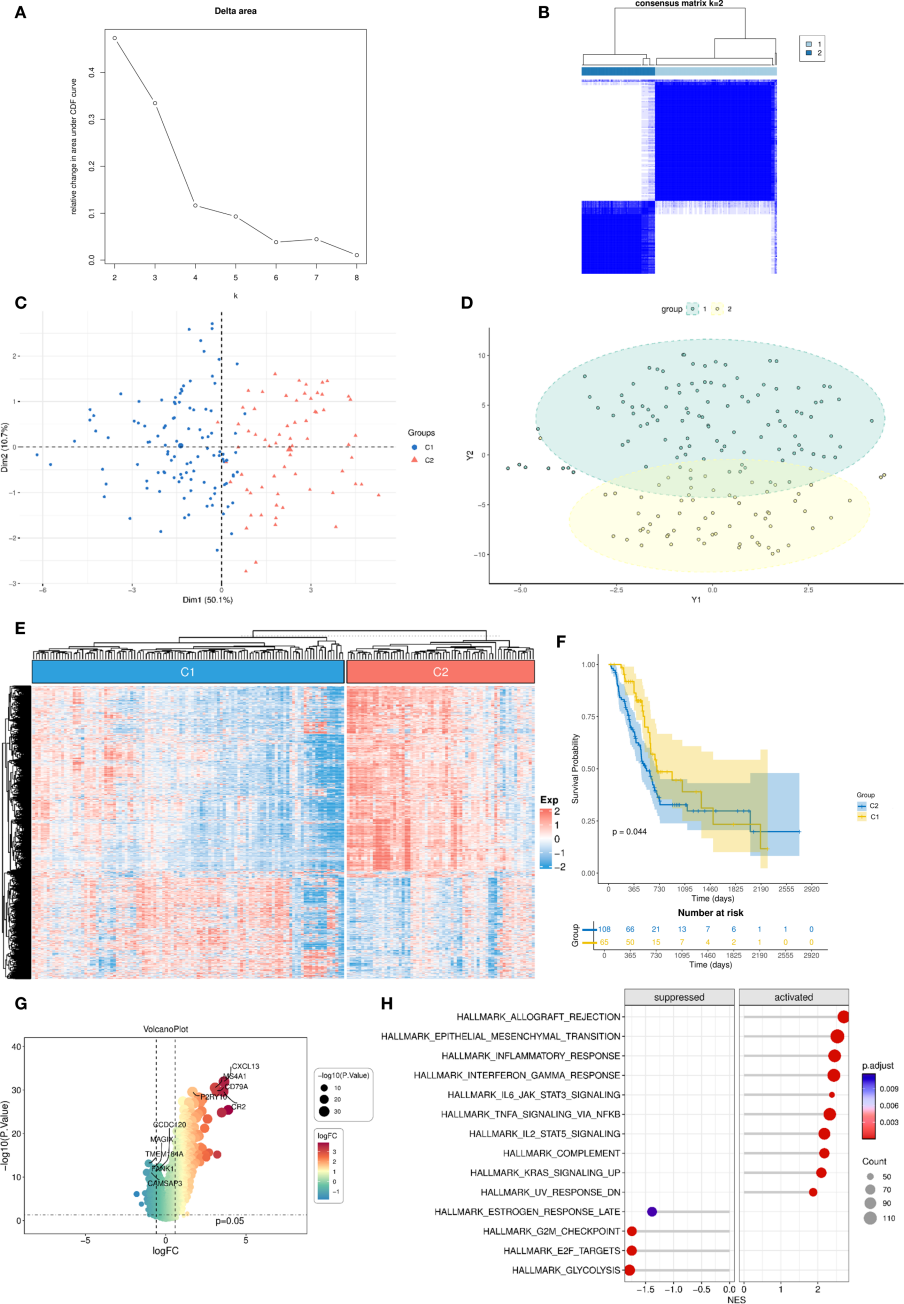
Unsupervised clustering into two DC–related clusters with expression profiling and survival analysis. **(A, B)** Consensus clustering analysis identifies two DC-related clusters in the TCGA-PAAD dataset; **(C, D)** PCA demonstrates distinct separation between the two DC-related clusters; **(E)** Heatmap shows the enrichment levels of DC-related genes in the two clusters; **(F)** Kaplan-Meier curve of overall survival between the two DC-related clusters. **(G)** Volcano plot of differentially expressed genes between the two DC-related clusters; **(H)** GSEA analysis of differentially expressed genes. DC, dendritic cell; TCGA, The Cancer Genome Atlas; PAAD, pancreatic adenocarcinoma; PCA, principal component analysis; GSEA, Gene Set Enrichment Analysis.

Using unsupervised consensus clustering based on the 130 DC–related genes, we stratified TCGA-PAAD patients into two distinct molecular clusters (**Figures 2A, B**). Principal component analysis confirmed clear separation between them (**Figures 2C, D**). The heatmap demonstrated generally higher expression of DC–related genes in cluster 2 compared to cluster 1 (**Figure 2E**). Survival analysis revealed that patients classified into cluster 1 demonstrated significantly improved overall survival (OS) compared to those in cluster 2 (*P* = 0.044, **Figure 2F**). Differential expression analysis between the two clusters identified 1,827 DEGs (adjusted *P* < 0.05, |log_2_FC| > 1, **Figure 2G**). Subsequent GSEA indicated that these DEGs were particularly enriched in immune-associated pathways, such as IL6–JAK–STAT3 signaling, TNF-α signaling via NF-κB, and IL2–STAT5 signaling, underscoring functional divergence in immune regulation between the two clusters (**Figure 2H**).

### 3.3 Construction and validation of a DC–related prognostic signature

**Figure 3:**
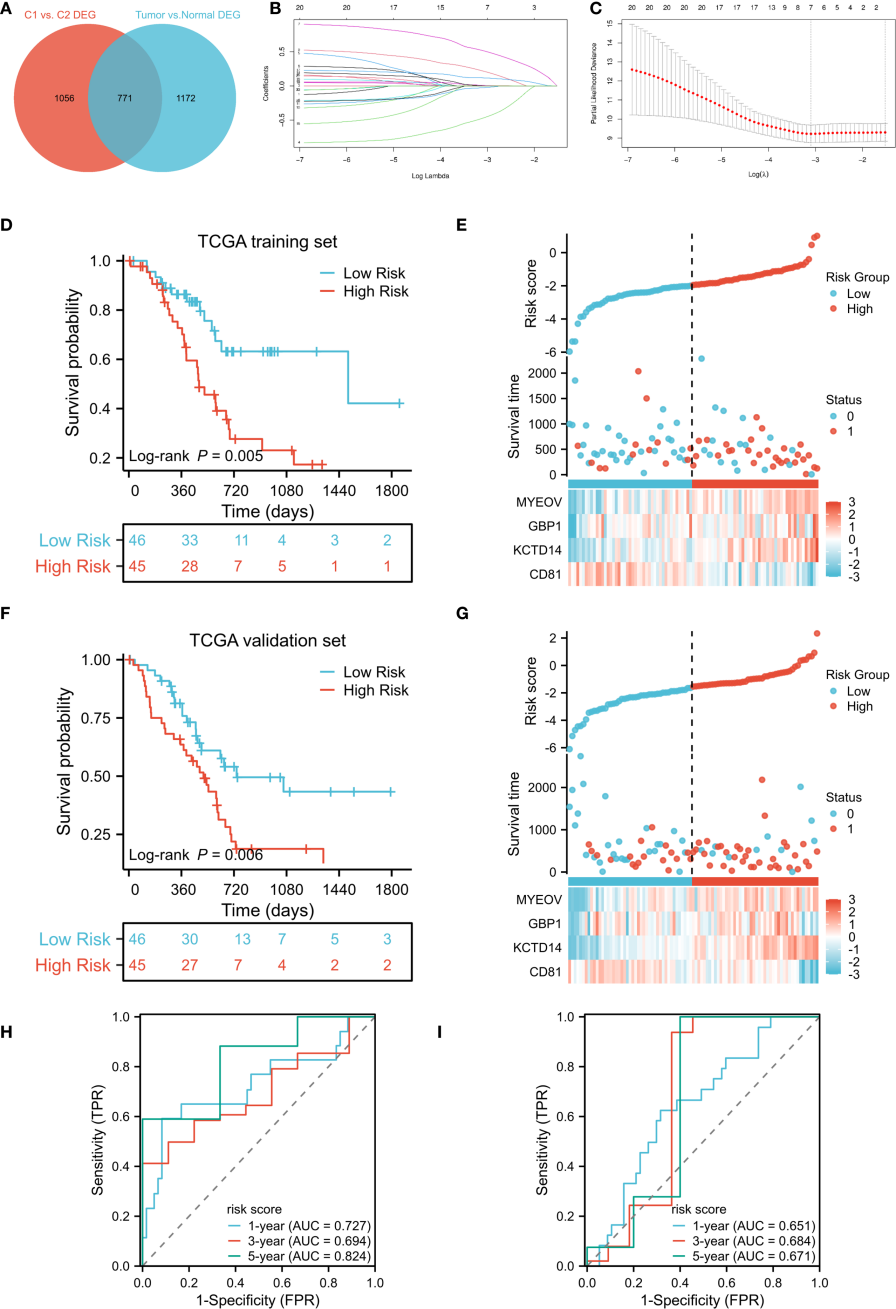
Development and validation of a DC-related gene signature. **(A)** Venn diagram identifies the intersection of DEGs in DC-related clusters and DEGs between TCGA-PAAD tumor and adjacent non-tumor tissues; **(B, C)** Construction of the DC-related signature based on LASSO regression; **(D, F)** Kaplan–Meier curves of OS for DCRS risk groups in the TCGA training and TCGA validation cohort; **(E, G)** Distribution of risk scores and corresponding survival status in the TCGA training and TCGA validation cohort; **(H, I)** Time-dependent ROC curves of OS for the risk scores in the TCGA training and TCGA validation cohort. DC, dendritic cell; DEGs, differentially expressed genes; TCGA, The Cancer Genome Atlas; PAAD, pancreatic adenocarcinoma; LASSO, Least Absolute Shrinkage and Selection Operator; OS, overall survival; DCRS, DC related risk score; ROC, receiver operating characteristic.

To construct a prognostic model, we intersected the 1,827 cluster–associated DEGs with the 1,943 DEGs identified between tumor and adjacent normal tissues in TCGA-PAAD, yielding 771 candidate genes (**Figure 3A**). The TCGA cohort was randomly split into equal training and validation sets. In the training set, univariate Cox regression identified 22 genes significantly associated with OS (*P* < 0.05, **Supplementary Figure S2**). These candidates were further narrowed down using LASSO regression and stepwise regression analysis, resulting in a four-gene prognostic signature (**Figures 3B, C**). The DCRS was calculated using the formula: Risk score = *CD81* × -0.806289828 + *KCTD14* × 0.739669347 + *GBP1* × 0.415429681 + *MYEOV* × 0.17556951. Using the medium value of the risk score, patients were divided into high- and low-risk groups. In the TCGA training cohort, patients in the low-risk group demonstrated significantly better OS compared to the high-risk group (*P* = 0.005, **Figure 3D**). The risk curves also showed higher survival probability in the low-risk group (**Figure 3E**). Similarly, in the TCGA validation cohort, the low-risk group showed significantly better OS compare to high-risk group (*P* = 0.006, **Figure 3F**), with risk curves corroborating superior survival probability in the low-risk group (**Figure 3G**). Time-dependent ROC curves demonstrated that area under the curve (AUC) values of the DCRS for OS in the TCGA training cohort at 1-, 3-, and 5-year were 0.727, 0.694, and 0.824, respectively (**Figure 3H**). Despite a slight decrease in the AUC values of the TCGA validation cohort, the results also demonstrated relatively good predictive potential for the short- and long-term OS of PC patients (1-year:0.651; 3-year: 0.684; 5-year:0.671, **Figure 3I**).

### 3.4 Single-cell transcriptomic profiling of DC–related genes

**Figure 4:**
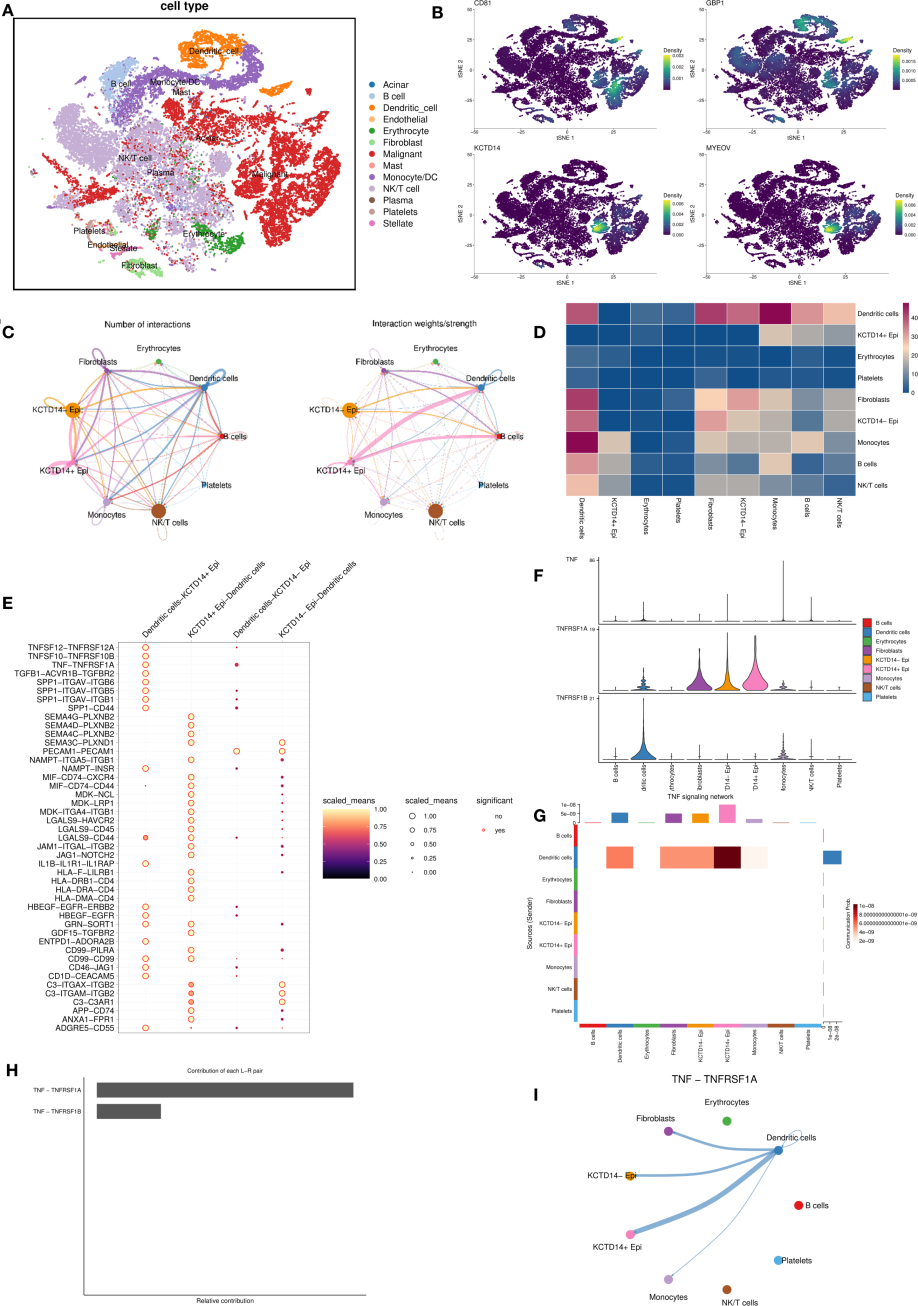
Single−cell transcriptomic profiling of DC-related gene expression and cellular communication. **(A)** Cell type annotation identifies 13 major cell clusters in the pancreatic cancer tumor microenvironment; **(B)** The expression of four key genes across the identified cell clusters; **(C, D)** Cell-cell communication analysis between epithelial cells with different KCDT14 expression status and various immune cell populations; **(E–I)** Pathway-specific analysis of epithelial cells with different KCDT14 expression status and DCs. DC, dendritic cell.

To elucidate cellular sources of the four signature genes, we analyzed single-cell RNA-seq data from PDAC tumors, clustering cells into 23 populations (**Supplementary Figure S3**). Cell type annotation identified 13 major cell clusters, including acinar cells, B cells, DCs, endothelial cells, erythrocytes, fibroblasts, malignant cells, mast cells, monocytes, NK/T cells, plasma cells, platelets, and stellate cells (**Figure 4A**). *CD81*, *KCTD14*, and *MYEOV* were predominantly expressed in malignant clusters, whereas *GBP1* localized primarily to DC clusters (**Figure 4B**).

### 3.5 Cell–cell communication analysis and pathway-specific analysis of *KCDT14*

In the risk model, *KCTD14* carried the largest positive coefficient, suggesting the strongest prognostic weight. *CD81* is a canonical exosomal tetraspanin involved in extracellular vesicles (EV) biogenesis and immune modulation across solid tumors. PDAC EV studies indicate that circulating EV features correlate with survival or treatment response, underscoring a biomarker-oriented role rather than a single-gene driver in tumor–DC crosstalk (25–27). *GBP1*, a prototypical IFN-induced GTPase, is enriched in myeloid/immune cells and mediates innate immune programs (28, 29). In line with this biology, our single-cell analysis localized *GBP1* predominantly to DC clusters rather than malignant epithelial clusters. In contrast, *MYEOV* already has PDAC-specific mechanistic evidence showing it enhances SOX9 transactivity to elevate HES1 and drive tumor progression (30), which reduces incremental novelty for the present study’s mechanistic focus. Therefore, we centered subsequent analyses on *KCTD14* in PDAC. The cell–cell communication analysis revealed significant negative correlations between *KCDT14* ^+^ epithelial cells and DCs (**Figures 4C, D**). Pathway-specific analyses showed that the connection between *KCDT14* ^+^ epithelial cells and DCs is related to a series of signaling pathways, including TNF-TNFRSF1A, SPPA-CD44, and SEMA4G-PLXNB2 (**Figure 4E**). Given that prior GSEA, GO, and KEGG results consistently highlighted TNF signaling among DC-related gene programs (**Figures 1H, I**; **Figure 2H**), we prioritized TNF pathway for in-depth investigation. The results confirmed that the TNF-TNFRSF1A pathway plays a crucial role in the connection between *KCDT14* ^+^ epithelial cells and DCs (**Figures 4F–I**).

### 3.6 *In vitro* validation of KCTD14 function in PC cell lines

**Figure 5:**
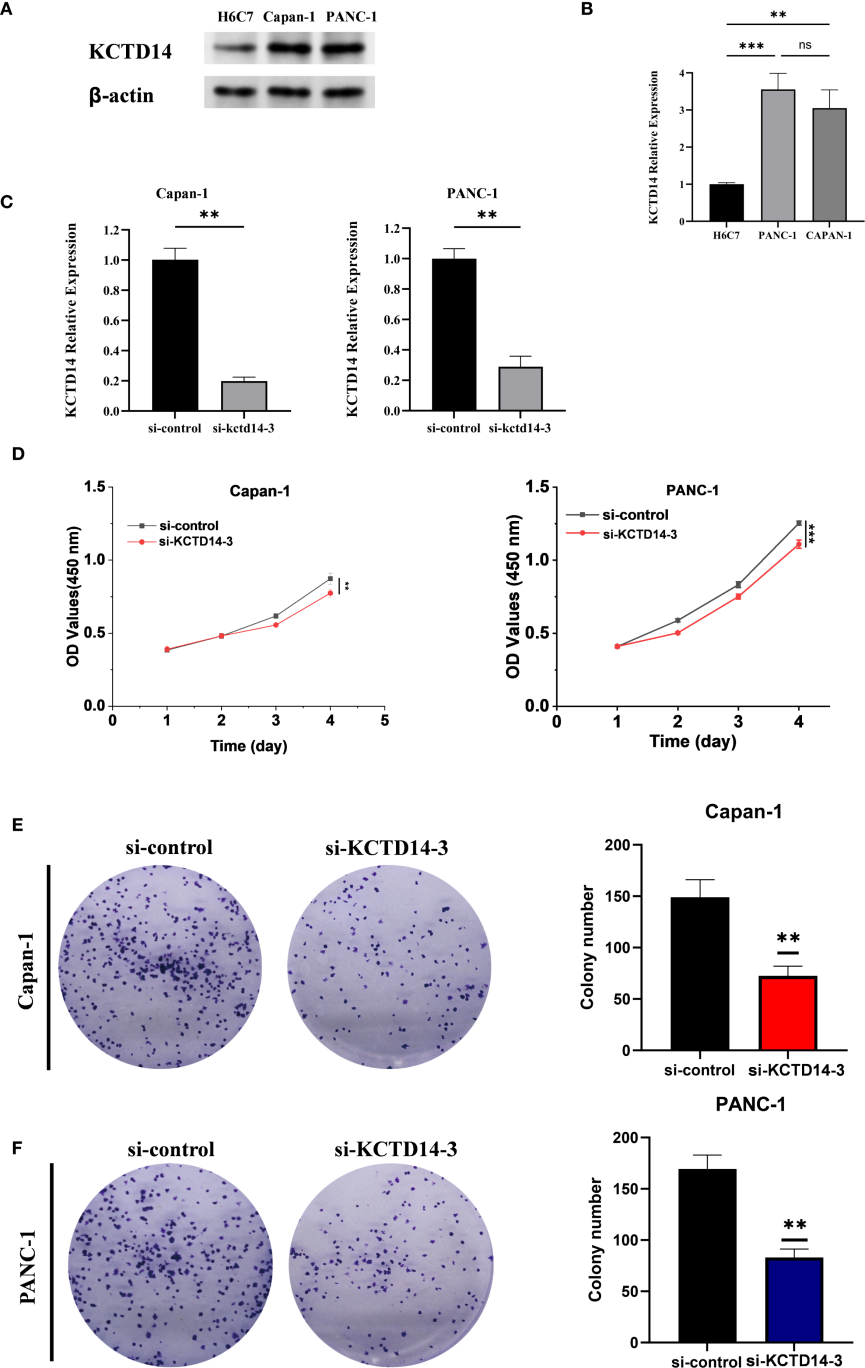
*In vitro* validation of *KCTD14* knockdown effects on proliferation and colony formation in pancreatic cancer cell lines. **(A, B)** Baseline *KCTD14* mRNA and protein levels are significantly higher in CAPAN-1 and PANC-1 cells compared to the H6C7 cell line (**P < 0.01, ***P < 0.001); **(C)** *KCTD14* expression is significantly reduced in both the CAPAN-1 and PANC-1 cell lines after knockdown (**P < 0.01); **(D)** CCK-8 assays show significantly reduced viability of CAPAN-1 and PANC-1 cells following *KCTD14* knockdown (**P < 0.01, ***P < 0.001); **(C, D)** Colony formation assays demonstrate a significantly decrease in clonogenic capacity after *KCTD14* knockdown in CAPAN-1 and PANC-1 cell lines (**P < 0.01).

**Figure 6:**
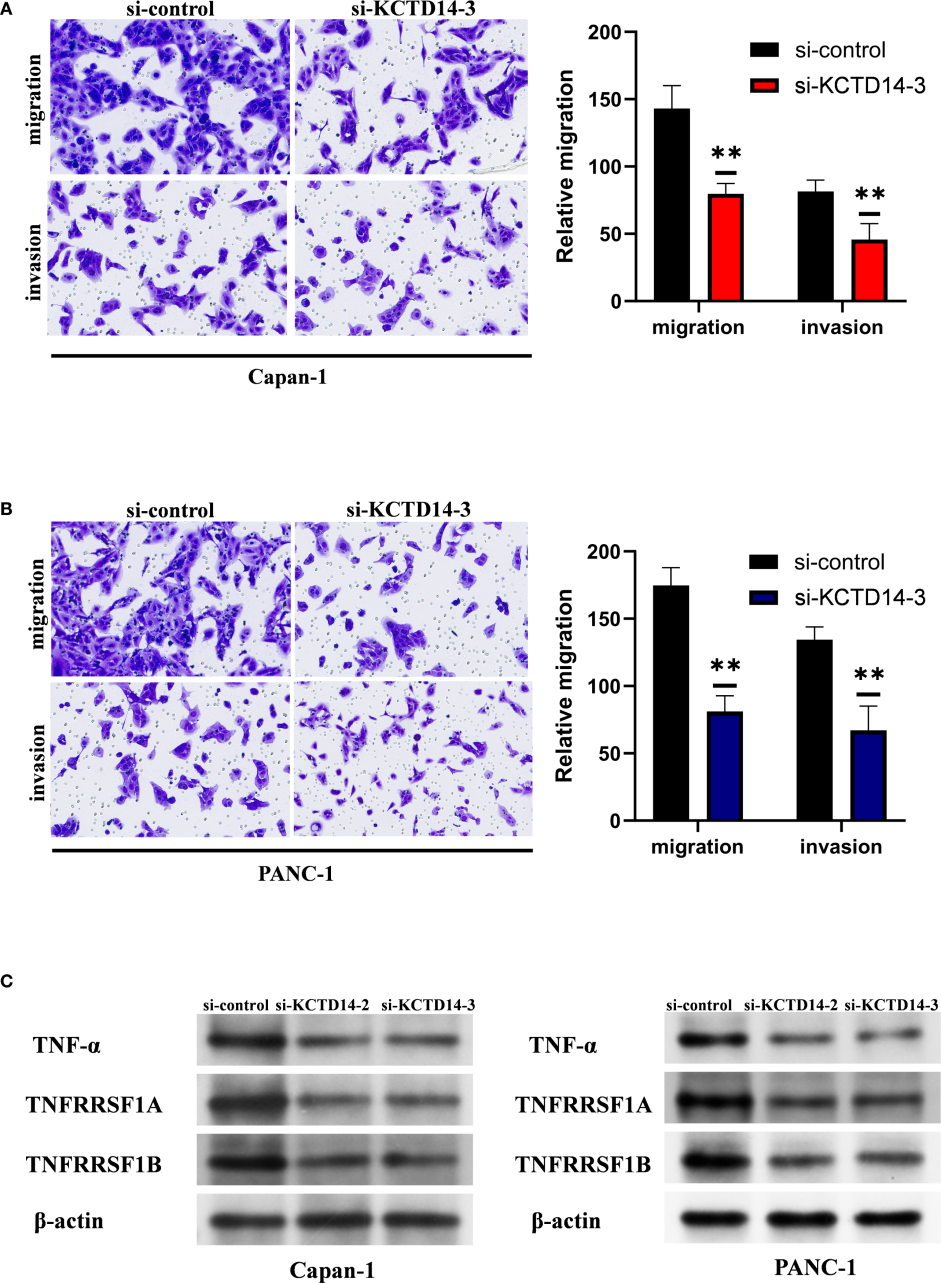
*KCTD14* knockdown significantly suppresses migration, invasion, and TNF-TNFR1 signaling in pancreatic cancer cell lines. **(A, B)** Transwell assays reveal significantly reduced migration and invasion abilities of CAPAN−1 and PANC−1 cells after *KCTD14* knockdown (**P < 0.01). **(C)** Western Blot analysis shows decreased expression of TNF-α, TNFRSF1A, and TNFRSF1B in *KCTD14*-knockdown cells compared to controls.

*KCTD14* expression and function were validated in H6C7, CAPAN-1, and PANC-1 cell lines. According to the Western Blot and qRT-PCR assays, the expression of *KCTD14* was significantly up-regulated in CAPAN-1 and PANC-1 cell lines compared with H6C7 cell line (**Figures 5A, B**, **P < 0.01, ***P < 0.001). Among three siRNAs, si-KCTD14–3 achieved the highest knockdown efficiency and was therefore chosen for subsequent experiments. In both CAPAN-1 and PANC-1 cell lines, the expression of *KCTD14* was significantly reduced after si-KCTD14–3 transfection (**Figure 5C**, **P < 0.01). Functionally, the cell viability was significantly reduced following *KCTD14* knockdown in both cell lines (**Figure 5D**, **P < 0.01, ***P < 0.001). Clonal formation assays demonstrated significantly fewer colonies in both cell lines after *KCTD14* knockdown (**Figures 5E, F**, **P < 0.01). In Transwell assays, the numbers of migrated and invaded cells significantly decreased in both cell lines following KCTD14 knockdown (**Figures 6A, B**, **P < 0.01). The Western Blot results confirmed lowered protein levels of TNF-α, TNFRRSF1A, and TNFRRSF1B following *KCTD14* knockdown in both cell lines (**Figure 6C**). Collectively, *KCTD14* knockdown significantly reduced PC cell proliferation, colony formation, migration, and invasion, and downregulated TNF pathway–related proteins.

## 4 Discussion

PC remains one of the most lethal malignancies, in part due to its profoundly immunosuppressive tumor microenvironment (TME) and the paucity of reliable prognostic biomarkers (31–33). Here, we leveraged WGCNA across three independent transcriptomic cohorts to uncover a DC-related gene set that captures inter-tumoral heterogeneity in immune infiltration. Consensus clustering based on these 130 DC-related genes robustly stratified patients into two subgroups with distinct OS, underscoring the central role of DCs in shaping PC prognosis.

Building upon these findings, we developed a four-gene DCRS via Cox and LASSO regression analysis. The DCRS demonstrated consistent prognostic power of OS in both training and validation cohorts, with AUC values exceeding 0.65 at 1-, 3-, and 5-year time points. Notably, high DCRS scores correlated with poorer OS, aligning with prior reports of similar signatures in hepatocellular carcinoma and colorectal cancer (34, 35). In addition, previous PDAC signatures have primarily captured T cell–inflamed states, delineated broad molecular subtypes, or linked epigenetic programs to immunity through methylation-based features (6, 7, 36). While these gene signatures are prognostic and valuable for stratification, they are not DC-centric and typically do not converge on a tumor-intrinsic, targetable pathway that explains DC loss. In contrast, our DCRS advances beyond prior PDAC signatures by being derived directly from DC-associated modules, integrating single-cell communication analyses to explore a tumor-intrinsic KCTD14–TNFR1 axis, and aligning with PDAC-specific preclinical evidence on DC depletion and the therapeutic potential of TNFR1 blockade (9). Collectively, this positions DCRS not only as a prognostic biomarker but also as a mechanistically actionable signature.

In the present study, the DCRS also captures a DC–anchored program that influences broader antitumor immunity. First, cDC1–T-cell coupling depends on effective DC recruitment and cross-priming. Across tumor settings, NK cells recruit cDC1 via CCL5/XCL1–XCR1, and disruption of this circuit contributes to immune exclusion (37). In PDAC, the paucity of DCs is a causal determinant of immune failure, whereas restoring DC abundance and function reinstates T-cell priming (12). Moreover, myeloid reprogramming can complement DC recovery. For example, CD40 agonists reprogram macrophages, remodel the stroma, and enhance DC costimulatory capacity (MHC-II, CD80, CD86), thereby facilitating antigen trafficking and T-cell activation (38, 39).

A particularly novel insight from our study is the identification of *KCTD14* as a pivotal mediator of DC-tumor crosstalk in PDAC. *KCTD14*, a member of the potassium channel tetramerization domain family, has been found to be associated with cancer hallmarks and involved in the modulation of specific oncogenic pathways (40–42). Our single-cell RNA-seq analyses revealed enriched *KCTD14* expression in malignant epithelial cells and a strong association with the low-immune (poor prognosis) cluster. The cell-cell communication and pathway specific analyses further implicated *KCTD14* in signaling to DCs via the TNF–TNFRSF1A (TNFR1) axis. Chronic TNF signaling through TNFR1 can induce DC apoptosis and promote immune escape in PDAC (9). In line with this, targeting TNF^+^ myeloid programs in PDAC can activate antitumor immunity and expand DC/T-cell activity, further reinforcing the biological plausibility of a TNF-centered axis in this disease (43). Therefore, it is plausible that tumor-derived *KCTD14* modulates TNF availability or receptor activation, thereby exacerbating DC depletion. Many KCTDs act as CUL3 E3-ligase adaptors via their BTB domains and can tune NF-κB pathway components. Some KCTDs modulate signaling by scaffolding/ubiquitinating receptors or adaptors (40, 44). We therefore hypothesize KCTD14 may scaffold CUL3-dependent ubiquitination that stabilizes TNF/TNFR1 signaling complexes, or alter receptor internalization/turnover, thereby biasing downstream TNFR1–NF-κB outputs that deplete DCs in PDAC. Functional assays confirmed that *KCTD14* knockdown in PDAC cells significantly reduced proliferation, migration, and invasion, and diminished TNF-α/TNFRSF1A expression *in vitro*, supporting an oncogenic and immunomodulatory role.

Furthermore, our results reinforce the therapeutic relevance of targeting the TNF-TNFR1 pathway in PC. Preclinical evidence has demonstrated that selective TNFR1 inhibition can restore DC viability, augment T-cell priming, and synergize with checkpoint blockade more effectively than broad DC activation strategies (9, 45). The convergence of our KCTD14-TNF findings with these data suggested that combining *KCTD14* inhibition or downstream *TNFR1* blockade with existing immunotherapies may yield enhanced antitumor efficacy. Moreover, the repurposing of clinically approved TNF inhibitors or the development of TNFR1-specific antagonists could accelerate clinical translation and broaden therapeutic options. In parallel, additional approaches to bolster DC recovery and function merit consideration. Flt3L has been shown to expand cDC populations. When combined with CD40 agonism, Flt3L could promote DC maturation and T-cell priming. Recent translational data support feasibility and activity of Flt3L plus CD40 agonist combinations in PDAC (46). In addition, targeting the CXCL12–CXCR4 axis could overcome stromal and chemokine barriers, thereby enhancing T-cell infiltration and antitumor responses in PDAC, and potentially synergizing with the DC restoration captured by DCRS (47). Collectively, these therapeutic strategies highlight the potential of integrating TNFR1 blockade with DC-directed interventions to overcome immune resistance and improve outcomes in PC.

Despite these promising observations, there are still some limitations in the present study. First, our analysis is retrospective and based on publicly available transcriptomic datasets, where prospective clinical validation is necessary to confirm the utility of the DCRS as a prognostic tool. Second, although single-cell RNA sequencing data were analyzed, the majority of our findings rely on bulk RNA sequencing, which inherently averages signals across heterogeneous tumor samples and may obscure cell type–specific contributions or introduce confounding factors. Third, the mechanistic role of *KCTD14*, while supported by *in vitro* experiments, requires *in vivo* validation to establish causality. Integrating spatial transcriptomics and multiplex immunostaining into future prospective cohorts will enable high-resolution mapping of immune cell infiltration and direct visualization of DC–tumor interactions within the intact tissue microenvironment. Such approaches will be crucial for refining the DCRS, confirming its translational applicability, and informing rational design of combination immunotherapies in PDAC.

## 5 Conclusion

In summary, our study identified a novel DC-related gene signature that stratifies PC patients by prognosis and highlights KCTD14 as a novel immunomodulatory oncogene acting through the TNF-TNFR1 axis. Our findings provide a foundation for integrating DCRS into clinical risk assessment and for pursuing KCTD14/TNFR1-targeted therapies to overcome DC-mediated immune suppression in pancreatic cancer.

## Data Availability

The datasets analyzed in this study are publicly available from the following repositories: The Cancer Genome Atlas (TCGA), accessible via project ID TCGA-PAAD, and Gene Expression Omnibus (GEO), accession numbers: GSE62165, GSE85916, GSE242230 (single-cell RNA-seq data of PDAC). All data used are open-access and can be freely retrieved using the accession numbers provided above.
