# Supplementary material for: Dendritic cell–related gene signature in pancreatic cancer stratifies patient subtypes and implicates a KCTD14–TNF signaling axis
Source: Front Immunol. 2025 Sep 25;16:1665906. doi: 10.3389/fimmu.2025.1665906 (PMC12507779; doi:10.3389/fimmu.2025.1665906)
Supplement: Supplementary file 1 [file DataSheet1.docx]

**Supplemental Figures**

**Figure S1** Protein-protein interaction network construction identifies hub gene nodes of the dendritic cell-related genes.


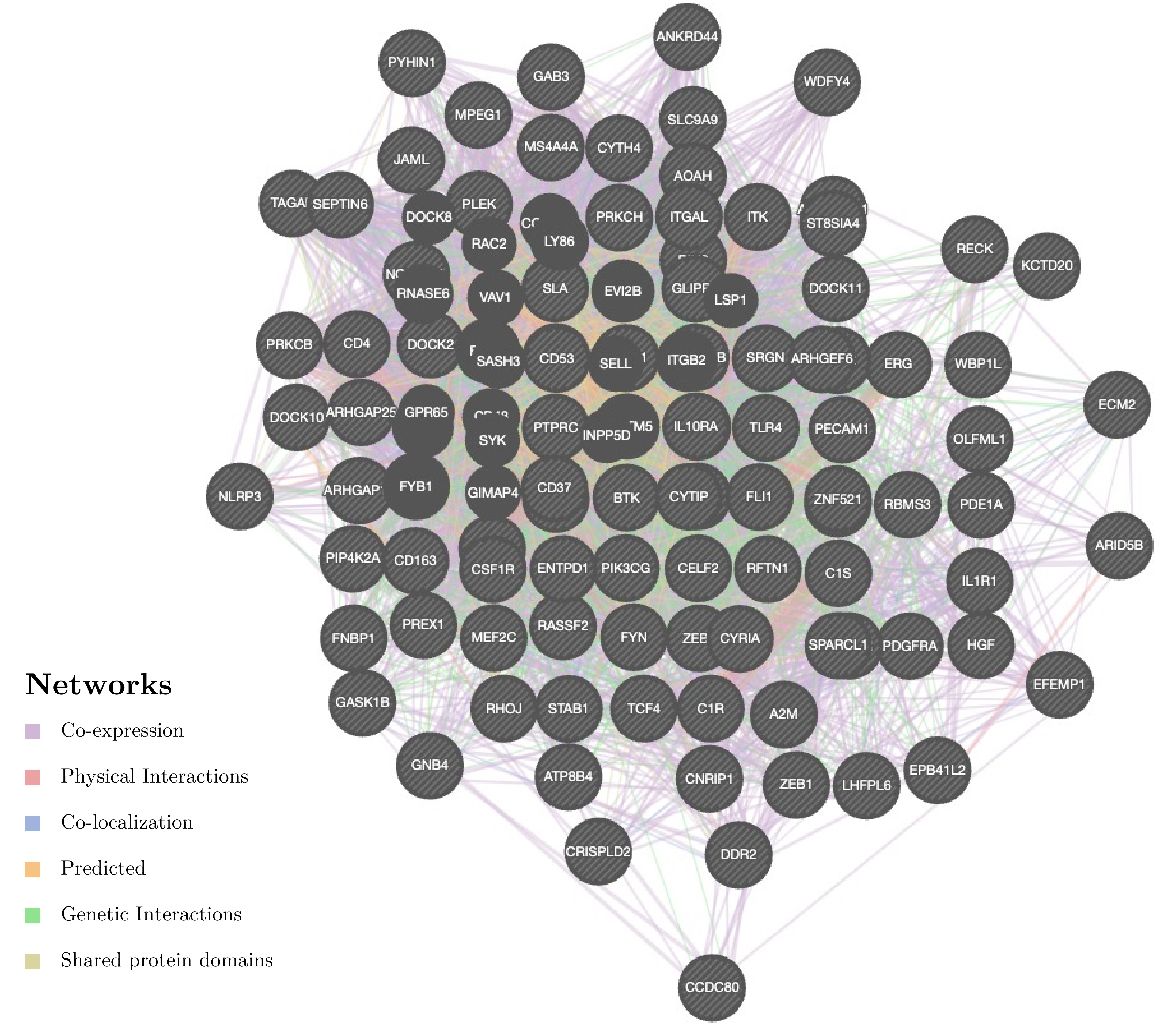


**Figure S2** Forest plot demonstrates that the univariate Cox regression identified 22 genes significantly associated with overall survival in the TCGA-PDAC training Cohort.
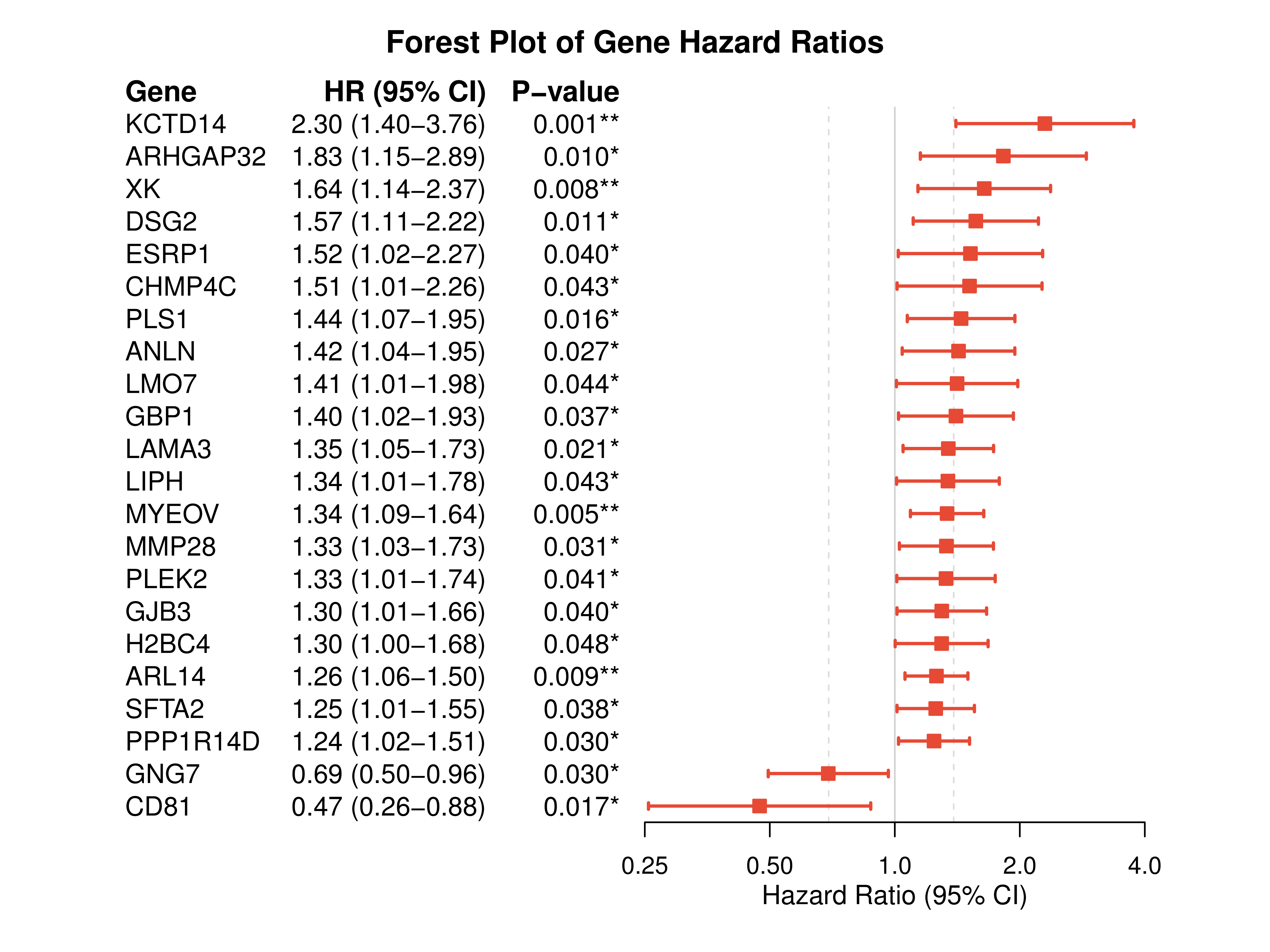


**Abbreviations:** TCGA, The Cancer Genome Atlas; PDAC, pancreatic ductal adenocarcinoma.

**Figure S3** Cell clustering analysis processes and clusters single-cell RNA seq data of PDAC tumors in GEO database into 23 populations.


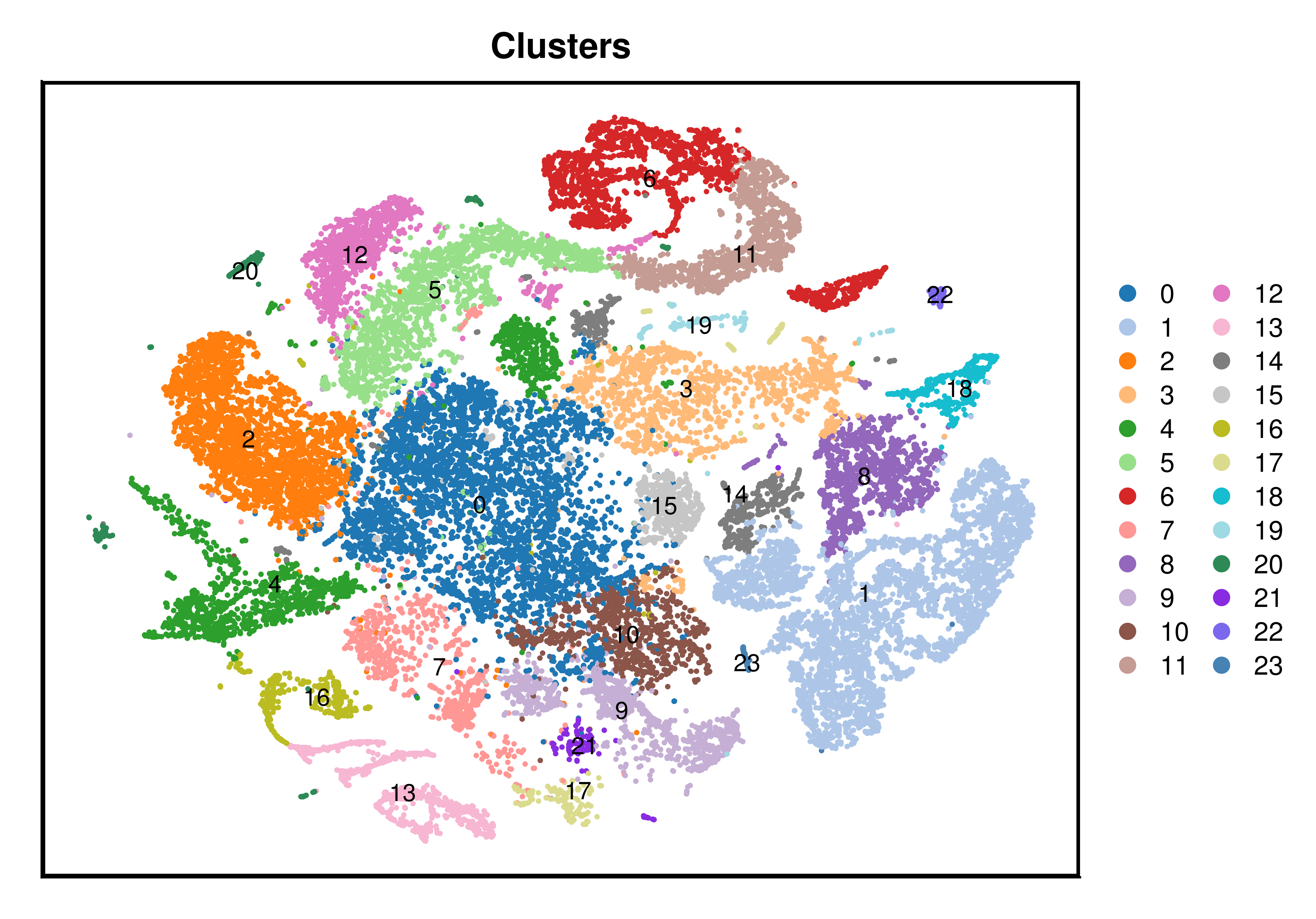


**Abbreviations:** PDAC, pancreatic ductal adenocarcinoma; GEO, Gene Expression Omnibus.
